# The Effects of Sodium Phosphate Supplementation on the Cardiorespiratory System and Gross Efficiency during Exercise under Hypoxia in Male Cyclists: A Randomized, Placebo-Controlled, Cross-Over Study

**DOI:** 10.3390/nu13103556

**Published:** 2021-10-11

**Authors:** Kamila Płoszczyca, Robert Gajda, Miłosz Czuba

**Affiliations:** 1 Department of Kinesiology, Institute of Sport, 01-982 Warsaw, Poland; milosz.czuba@insp.waw.pl; 2 Center for Sports Cardiology, Gajda-Med Medical Center, 06-100 Pułtusk, Poland; gajda@gajdamed.pl

**Keywords:** phosphate loading, ergogenic aids, hypoxia, altitude, cardiorespiratory system, myocardium, gross efficiency, athletes

## Abstract

The main aim of this study was to evaluate the effects of six days of tri-sodium phosphate (SP) supplementation on the cardiorespiratory system and gross efficiency (GE) during exercise under hypoxia in cyclists. Twenty trained male cyclists received SP (50 mg·kg^−1^ of fat-free mass/day) or placebo for six days in a randomized, cross-over study, with a three-week washout period between supplementation phases. Before and after each supplementation phase, the subjects performed an incremental exercise test to exhaustion under normobaric hypoxia (FiO_2_ = 16%, ~2500 m). It was observed that short-term SP supplementation led to a decrease in heart rate, an increase in stroke volume, and an improvement in oxygen pulse (VO_2_/HR) during low and moderate-intensity exercise under hypoxia. These changes were accompanied by an increase in the serum inorganic phosphate level by 8.7% (*p* < 0.05). No significant changes were observed in serum calcium levels. GE at a given workload did not change significantly after SP supplementation. These results indicated that SP promotes improvements in the efficiency of the cardiorespiratory system during exercise in a hypoxic environment. Thus, SP supplementation may be beneficial for endurance exercise in hypoxia.

## 1. Introduction

Over the years, it has been suggested that supplementation with phosphate salts may have a positive effect on athletic performance [1]. In 2021, phosphate was classified by the Australian Institute of Sport as a substance for which the scientific evidence does not support a benefit amongst athletes or no research has been conducted to form an informed opinion about the substance [2]. Undoubtedly, more research is needed to determine the effectiveness of phosphate supplementation in athletes and to establish the best practice protocol in this regard. To date, several studies have supported the exercise performance benefits of phosphate salt supplementation in normoxic conditions. These studies showed an increase in maximal oxygen uptake [3,4,5,6,7,8] and anaerobic threshold [4,7,9], better time trial performance [10,11], and improvements of the repeated-sprint ability [11,12,13,14]. A number of mechanisms have been proposed to explain the ergogenic effects of phosphate loading, including increasing oxidative metabolism and adenosine triphosphate (ATP) synthesis, and also more rapid restoration of ATP and phosphocreatine in the muscles (through greater availability of extracellular and intracellular phosphates), an improvement of oxygen unloading in muscles by reducing Hb-O_2_ affinity (via increasing erythrocyte 2,3-diphosphoglycerate concentrations), increasing buffering capacity (via enhancing extracellular hydrogen phosphate concentration), and improving myocardial function [1].

The improvement in myocardial and cardiovascular responses to exercise following phosphate supplementation is explained by increased myocardial contractility as a result of an increase in the levels of cardiac cell ATP [9]. Myocardial contractility is one of the determinants of heart stroke volume (SV). When the force of myocyte contraction increases, the heart can eject more blood out into the vasculature, thus increasing the SV [15]. Several studies have shown that phosphate loading leads to an increase in SV [9] and a decrease in heart rate (HR) [6,7,16,17] at rest and during exercise in normoxia. These adaptations were accompanied by improved aerobic capacity and time trial performance in athletes [6,7,9]. Keeping in mind that the heart is dependent on aerobic metabolism to sustain contractile function, and that in hypoxic conditions it responds with a compensatory increase in cardiac output (Q) that further increases myocardial O_2_ demand [18], it can be presumed that an improvement in myocardial function following phosphate loading may also be beneficial in hypoxic conditions. However, there are currently no studies analyzing the effect of phosphate salts on exercise performance and cardiorespiratory system efficiency under hypoxia.

Hypoxic exposure leads to important changes in the cardiovascular system [19]. During acute hypoxia, resting SV is unchanged, but HR increases through stimulation of the cardiac β-adrenergic receptors and an increase in circulating epinephrine, resulting in higher Q [20]. In contrast, maximal HR and SV are lowered and thus maximal Q declines in hypoxic conditions [21]. During submaximal exercise, at a given exercise intensity (at the same absolute workload), HR is greater and SV is lower in hypoxia compared to normoxia [19,20,22]. For example, Clark et al. [23] found that submaximal HR during 50–250 W workloads was significantly elevated by a mean of 5–21 bpm at both 2200 m and 3200 m compared to sea level. In other words, at the same HR, exercise capacity is lower in hypoxia than in normoxia. Recently, we observed that mean power generated by cyclists during a 30 km time trial in acute hypoxia (FiO_2_ = 16.5%, 2000 m) was 9.6% lower with the same average HR as in normoxia [24]. Similarly, Weavil et al. [25] reported a 9% decrease in average power during a five km cycling time trial in hypoxia (FiO_2_ = 17%), while HR did not differ from that in normoxia.

Achieving adequate Q during exercise to ensure sufficient oxygen uptake for a given workload is determined by the changes in SV and HR [26]. In terms of myocardial oxygen demand, increasing SV is much more efficient than increasing HR during exercise [27]. Reduction of myocardial oxygen demand should be particularly preferred during exercise in hypoxic conditions when oxygen availability is limited. Therefore, the first aim of this study was to investigate the effect of six-day sodium phosphate (SP) supplementation on cardiorespiratory variables in cyclists during exercise in normobaric hypoxia (FiO_2_ = 16.0%; ~2500 m). We hypothesize that phosphate salt loading improves the efficiency of the cardiorespiratory system.

The second purpose of this study was to analyze whether SP supplementation would change gross mechanical efficiency (GE). GE is one of the main factors determining exercise performance [28,29]. Mechanical efficiency refers to the ability to transfer the energy expended into performing external work [30]. The increase in efficiency is associated with the improved conversion of chemical energy from ATP hydrolysis into mechanical energy during muscle contraction [28]. Less efficiency for a given work output is attributed to the higher energy cost of exercise [31,32]. Hypoxic conditions may decrease mechanical efficiency, but the results of previous studies are still inconclusive [23,33,34]. Noordhof et al. [35] proposed that the reduction of GE under hypoxic conditions could be caused by an increased cost of ventilation and HR in combination with a higher respiratory-exchange ratio (RER) during exercise.

It was demonstrated that the intracellular concentration of inorganic phosphate (Pi) is an important determinant of mitochondrial oxidative phosphorylation [36,37], and that a decrease in muscle ATP synthesis may be associated with low blood Pi concentrations [37]. Recently, Marcos et al. [38] reported that sodium phosphate intake improves energy efficiency (reduction of oxygen consumption at the same workload) during exercise at low intensity in normoxia. Thus, we expect that SP supplementation improves GE under hypoxic conditions.

## 2. Materials and Methods

### 2.1. Study Participants

Twenty trained male cyclists (aged 34.6 ± 4.3 years; body height 180.5 ± 5.9 cm; body mass 73.2 ± 6.6 kg; fat content (%) 13.9 ± 3.4%; fat-free mass (FFM) 63.0 ± 5.8 kg) participated in this study. The basic inclusion criteria were: training experience of at least six years and at least a six-month wash-out period without altitude training and sodium phosphate supplementation. Furthermore, before starting the experiment, blood ion concentrations were analyzed in all participants under fasting conditions. All athletes had the correct blood ion levels before the experiment. All athletes had current medical examinations without any contraindications to performing exhaustive exercise in a hypoxic environment. The participants provided their written voluntary informed consent before participation. Additionally, participants declared that for at least one month before testing, they did not take either medications or dietary supplements. Study participants were randomly divided into two equal research groups, G1 and G2, using a computer-generated randomized list [39].

The research project was conducted according to the Declaration of Helsinki and was approved (no. 4/2018, approval date: 15 November 2018) by the Ethics Committee for Scientific Research at the Jerzy Kukuczka Academy of Physical Education in Katowice, Poland.

### 2.2. Study Design

**Figure 1:**
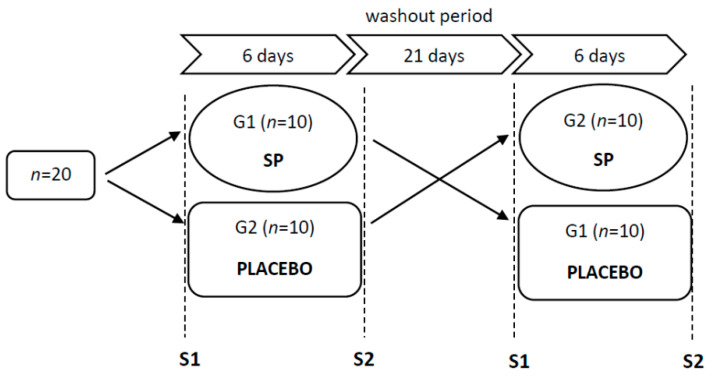
Study design. SP—sodium phosphate supplementation (50 mg/kg FFM per day); S1 and S2—two test series, before and after each supplementation phase.

The study was carried out in a cross-over design (Figure 1). The experiment included two six-day supplementation phases with a three-week washout period (for details, see in the “Supplementation with sodium phosphate” section). Before and after each supplementation phase, a test series (S1, S2) was performed. All series were based on the same methodology. The time of day and the order of the participants were maintained in order to ensure similar conditions for the measurements. Each test series began by obtaining venous blood (10 mL) from an antecubital vein under fasting conditions to determine resting levels of the following variables: hemoglobin concentration ([Hb]), hematocrit (Hct), red blood cell count (RBC), and serum levels of inorganic phosphate (Pi) and calcium (Ca). After blood had been obtained, body height, body mass, and body composition were evaluated using the DXA (dual-energy X-ray absorptiometry) method (GE Lunar Prodigy). Hematocrit value and anthropometric variables were used to calculate plasma volume (PV) according to the formula proposed by Nadler et al. [40].

Two hours after a light mixed meal (5 kcal/kg body weight, 50% CHO, 30% Fat, 20% Pro) and after 15 min of passive exposure to normobaric hypoxia (FiO_2_ = 16%; ~2500 m), all participants performed an incremental exercise test under normobaric hypoxia to determine the values of VO_2max_ and lactate threshold (LT). The incremental test was performed using the Excalibur Sport cycle ergometer (Lode, Netherlands). Exercise tests were performed in a normobaric hypoxic chamber (AirZone 25, Air Sport, Poland). During all test series, the atmospheric conditions such as temperature (19 °C), humidity (50%), the concentration of carbon dioxide (700–800 ppm), and oxygen (FiO_2_ = 16%) were controlled and held constant to increase the reliability of the investigations.

### 2.3. Incremental Exercise Test

The same test protocol was applied for all exercise tests in each series of testing. The incremental test started with a load of 80 W which was increased by 40 W every three min to exhaustion or until the participant was unable to maintain the minimal cadence of 60 rpm. At rest (three min before the test) and during the exercise, heart rate (HR), minute ventilation (VE), breathing frequency (BF), oxygen uptake (VO_2_), expired carbon dioxide (VCO_2_) and respiratory exchange ratio (RER) were measured continuously with a fast gas analyzer (MetaLyzer 3B, Cortex) using the breath-by-breath method. At the end of each workload (last 15 s), capillary blood samples were drawn from the fingertips to determine blood lactate levels (SUPER GL2, Dr. Müller Gerätebau GmbH). These data were used to evaluate individual lactate threshold (LT) based on the D_max_ method [41]. Our previous studies [42,43] demonstrated that LT determined using the D-max method corresponds to the maximal lactate steady state (MLSS).

During the first test of each supplementation phase (S1), HR, VO_2_, oxygen pulse (VO_2_/HR), stroke volume (SV), and cardiac output (Q) at LT workload (LT), as well as one and two workloads below and above LT (LT−1, LT−2, LT+1, LT+2) were analyzed. During the next tests (S2), these variables were identified at the same absolute workloads as during the first test. Q was estimated by VO_2max_, and was calculated by the following formula: $Q=100\times \mathrm{VO}2÷\left(5.72+10.5a\frac{\mathrm{VO}2}{\mathrm{VO}2\max}\right)$ used by MetaSoft Studio (Cortex, Germany). SV was calculated as follows: SV=Q÷HR.

### 2.4. Supplementation with Sodium Phosphate

During 6 days of the supplementation phase, subjects from group G1 received tri-sodium phosphate at a dose of 50 mg/kg FFM per day. The dose was divided into equal portions and administered four times a day at similar intervals. The participants in group G2 received a placebo in the form of 4 g of cellulose per day, also at a dose divided into four equal portions. Study participants were not informed which substance they were taking. The first six-day supplementation phase was followed by a 21-day substance wash-out period during which participants were not supplemented. Next, the second supplementation phase began. It was identical to the first but the groups were changed so that the six-day supplementation with SP was used in group G2, whereas group G1 received a placebo. Throughout the experiment, all participants received nutritional, training and supplementation recommendations. 

SP dose (50 mg/kg FFM per day) and supplementation time (6 days) were selected based on the methodology used in previous studies on ergogenic effects of phosphate salt supplementation in normoxia [7,44,45,46]. Buck et al. [1] demonstrated that the dose of 3–5 g of SP per day applied over a period of 3–6 days is adequate to achieve sufficient serum phosphate levels and to ensure the expected supplementation benefits. Doses greater than 6 g are usually avoided during supplementation, as they are associated with a reduction in the serum phosphate concentration through regulation of its level through parathormone. Doses below 3 g are generally considered too low to significantly increase serum phosphate levels [1,47,48]. The duration of the washout period (21 days) was selected according to the suggestion that the washout time should be two to three weeks in order to remove any carryover effects from previous phosphate supplementation [3]. 

### 2.5. Determination of Gross Efficiency

GE was calculated as the ratio of work accomplished per minute (watts converted to kcal/min) to energy expended per minute (kcal/min). Energy expenditure was calculated from VO_2_ and RER using the tables of Lusk [49].

(1) $$\mathrm{GE}=\frac{\mathrm{Work}\;\mathrm{accomplished}}{\mathrm{Energy}\;\mathrm{expended}}\times 100\%$$

During the first test of each supplementation phase (S1) GE at one and two workloads below LT (LT−1, LT−2) was analyzed. During the next tests (S2), GE was identified at the same absolute workloads as during the first test. GE was only calculated when the RER was ≤1.0. The measured RER was converted to the caloric equivalent of oxygen for the non-protein respiratory quotient [49]. During exercise at LT and higher workloads, RER was >1.0, thus GE was calculated only for workloads below LT.

### 2.6. Statistical Analysis

The results of the study were analyzed using StatSoft Statistica 13.0 software. The results were presented as mean ±SEM. The statistical significance level was set at *p* < 0.05. Prior to all statistical analyses, normality of the distribution of variables was checked using the Shapiro-Wilk test. The analysis of variance (ANOVA) for repeated measures (intervention [SP, placebo] × time [S1,S2]) was used to determine differences in each of the dependent variables. When significant differences were found, the post hoc Tukey test was used. Effect sizes (ESs) were calculated from standardized differences (Cohen’s d units). Threshold values for Cohen ES statistics were considered to be small (0.20–0.60), moderate (0.60–1.20), large (1.20–2.0), very large (2.0–4.0), or extremely large (>4.0) [50].

## 3. Results

### 3.1. Exercise Intensity

The LT workload (LT), as well as one and two workloads below and above LT (LT−2, LT−1, LT+1, LT+2), corresponds to absolute workload determined during the initial exercise test (S1). The exercise intensity reported as a % of actual VO_2max_ measured during S1 and S2 is presented in Table 1. 

ANOVA with repeated measures showed a significant interaction (intervention x time) for % of actual VO_2max_ at two workloads above LT (F = 6.64, *p* < 0.05). The post-hoc Tukey’s test revealed a decrease in % of actual VO_2max_ at LT+2 workload (*p* < 0.05) following SP supplementation (Table 1). No significant changes were observed after placebo ingestion.

### 3.2. Cardiorespiratory Variables

HR, SV, Q, VO_2_ and VO_2_/HR were analyzed at workloads (LT−2, LT−1, LT, LT+1, LT+2) determined during the initial exercise test (S1). During the next test (S2), these variables were identified at the same absolute workloads as during the S1.

ANOVA with repeated measures showed a significant interaction (intervention x time) for HR at workloads below LT (HR_LT−2_: F = 3.83, *p* < 0.05; HR_LT−1_: F = 5.97, *p* < 0.05) and for HR at LT (HR_LT_: F = 4.90, *p* < 0.05). Statistically significant changes were also found for SV at LT and at one workload below LT (SV_LT−1_: F = 7.72, *p* < 0.01; SV_LT_: F = 5.98, *p* < 0.05). Furthermore, changes at the limit of the adopted significance level were observed for oxygen pulse at one workload below LT (VO_2_/HR_LT−1_; F = 3.64, *p* < 0.07). 

**Figure 2:**
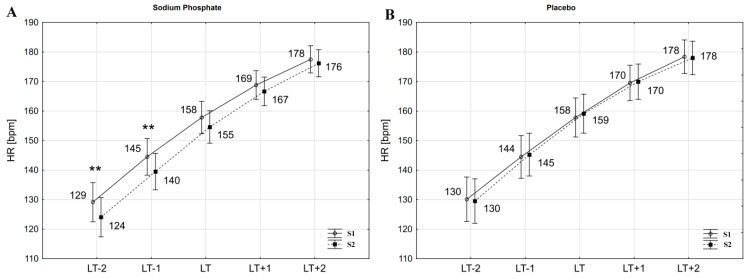
Heart rate (HR) during exercise in hypoxia before (S1) and after (S2) sodium phosphate (**A**) and placebo (**B**) ingestion. ** *p* < 0.01 S1 vs. S2. LT−2, LT−1, LT, LT+1, LT+2—absolute workload corresponding to workload at lactate threshold (LT) as well as one and two workloads below and above LT which were determined during the initial exercise test (S1).

**Figure 3:**
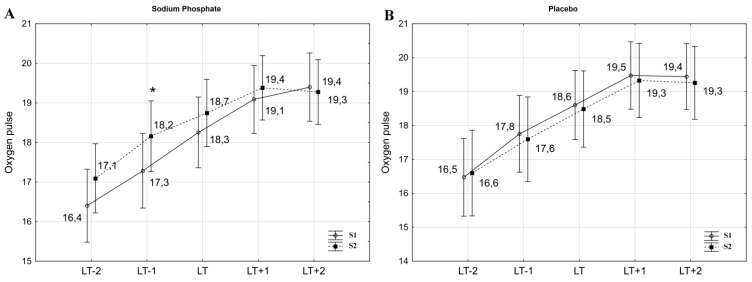
Oxygen pulse (VO_2_/HR) during exercise in hypoxia before (S1) and after (S2) sodium phosphate (**A**) and placebo (**B**) ingestion. * *p* < 0.05 S1 vs. S2. LT−2, LT−1, LT, LT+1, LT+2—absolute workload corresponding to workload at lactate threshold (LT) as well as one and two workloads below and above LT which were determined during the initial exercise test (S1).

The post-hoc Tukey test showed that HR_LT−2_ and HR_LT−1_ significantly (*p* < 0.01) decreased due to SP supplementation (Figure 2A) by 3.9% (d = 0.36) and 3.4% (d = 0.38), respectively. Additionally, HR_LT_ dropped by 1.9% following SP, although not significantly (*p* < 0.06, d = 0.27). Similar changes were not observed after placebo ingestion (Figure 2B). Analysis also revealed a significant increase in SV_LT−1_ by 4.1% (*p* < 0.01, d = 0.47) and in SV_LT_ by 2.5% (*p* < 0.05, d = 0.30). SV_LT−2_ increased by 3.7%, but these changes did not reach statistical significance (*p* < 0.08, d = 0.44) (Table 2). Furthermore, SP supplementation improved VO_2_/HR_LT−1_ by 5.2% (*p* < 0.05, d = 0.45) (Figure 3A). At workloads above LT, there were no significant changes in HR, SV and VO_2_/HR. VO_2_ at the given workloads also did not change significantly after the interventions (Table 2).

There were no statistically significant changes in cardiac output (Q) at the given workloads (Table 2). Q_max_ and SV_max_ were unchanged after SP supplementation (S1 vs. S2: SP 19.13 ± 0.29 vs. 19.01 ± 0.30 L∙min^−1^; Pl 19.23 ±0.31 vs. 19.23 ±0.32 L∙min^−1^ and SP 107.2 ± 2.2 vs. 107.0 ± 2.4 mL; Pl 106.2 ± 2.3 vs. 108.4 ±2.5 mL, respectively). Furthermore, VO_2max_ and HR_max_ also did not change significantly following the interventions (S1 vs. S2: SP 3.45 ± 0.06 vs. 3.51 ± 0.06 L∙min^−1^; Pl 3.47 ± 0.06 vs. 3.44 ± 0.06 L∙min^−1^ and SP 178.7 ± 2.4 vs. 178.1 ± 2.5 bpm; Pl 179.6 ± 2.6 vs. 179.4 ± 2.6 bpm, respectively).

### 3.3. Gross Efficiency

Gross efficiency (GE) and RER during S1 and S2 were analyzed at the same absolute workloads. There were no statistically significant changes in GE and RER at the given workloads after SP supplementation or placebo ingestion (Table 3).

### 3.4. Serum Phosphate and Calcium Concentrations

ANOVA with repeated measures showed a significant interaction (intervention x time) for serum Pi level (F = 4.16, *p* < 0.05). The post-hoc Tukey test revealed that Pi concentration increased by 8.7% (*p* < 0.05, d = 0.72) following the SP supplementation (S1 vs. S2: SP 3.11 ± 0.08 vs. 3.38 ± 0.09 mg/dL; Pl 3.32 ± 0.08 vs. 3.33 ± 0.09 mg/dL). No significant changes were observed in serum Ca level (S1 vs. S2: SP 9.55 ± 0.06 vs. 9.51 ± 0.06 mg/dL; Pl 9.53 ± 0.06 vs. 9.47 ± 0.06 mg/dL).

### 3.5. Hematocrit and Plasma Volume

There were no statistically significant changes in Hct (S1 vs. S2: SP 43.6 ± 0.6 vs. 43.1 ± 0.5%; Pl 44.1 ± 0.5 vs. 43.3 ± 0.5%) and PV (S1 vs. S2: SP 2888 ± 58 vs. 2909 ± 58 mL; Pl 2831 ± 57 vs. 2872 ± 56 mL) after SP supplementation or placebo ingestion.

## 4. Discussion

According to the authors’ knowledge, this is the first study to analyze the ergogenic effects of phosphate salt supplementation on exercise performance under hypoxic conditions. Our study revealed that short-term SP supplementation leads to a decrease in HR, an increase in SV, and an improvement in oxygen pulse (VO_2_/HR) during exercise at low to moderate intensity (≤LT) under hypoxia (2500 m). These results indicated that SP promotes improvements in the efficiency of the myocardial and cardiorespiratory systems during exercise in a hypoxic environment. Thus, SP supplementation may be beneficial for endurance exercise performance in hypoxia. Lowering HR and increasing SV while maintaining the same ability to perform work during exercise reduces myocardial oxygen demand and may improve exercise tolerance.

Our findings are in line with the results of several previous studies conducted in normoxic conditions. It has been reported that phosphate loading caused a decrease in HR [6,7,16,17] and an increase in end-diastolic volume, SV and Q [9] during endurance exercise. In our previous studies [6,7] we found that after using the same supplementation protocol as in this study, the resting HR and HR at LT dropped by 9.6% and 1.7%, respectively, and maximal VO_2_/HR increased by 5.8% in elite mountain bike cyclists during an incremental exercise test in normoxia.

Notably, in our study a significant decrease in HR (by 5 bpm) and an improvement in VO_2_/HR (by 5.2%) following SP supplementation occurred only at workloads below the LT, where the exercise intensity was <75% VO_2max_. At LT workload, the reduction in HR was less pronounced (decrease by 3 bpm), and at higher intensities, no statistically significant changes in HR were found. Perhaps, as the exercise intensity increased above the anaerobic threshold, the stimulation of the sympathetic nervous system activity and an overproportional increase in release of catecholamines occurred [51,52]. Consequently, stimulation of the myocardium by the sympathetic nervous system resulted in an increase in HR [53,54]. We suggest that above LT, this mechanism was dominant in relation to the reduction in HR caused by the phosphate salts.

The basis for the assumption that ingestion of phosphate salts can contribute to improvement in myocardial efficiency are the observations that hypophosphatemia causes impaired contractility of the myocardium and reduced SV, while reversal of hypophosphatemic states has been reported to significantly improve the cardiac muscle work [55,56,57]. O’Connor et al. [55] reported that after intravenous infusion of potassium phosphate solution in patients with severe hypophosphatemia there was a significant increase in SV. The increase in SV was explained by an improvement in myocardial contractility. The authors suggested that the increase in myocardial contractility is most likely the result of an increase in intracellular ATP concentration, the level of which is low during hypophosphatemia. Recently, Pesta et al. [37] reported that ATP synthesis in muscle cells is markedly decreased in hypophosphatemic mice, suggesting that intramyocellular phosphate regulates ATP synthesis. Additionally, Brautbar and Altura [58] demonstrated that reduction in the myocardial concentrations of creatine phosphate and Pi is associated with a decline in the activity of mitochondrial and myofibrillar creatine phosphokinase, which plays a crucial role in skeletal and cardiac muscle contractility.

Davis et al. [59] reported that an improvement of ventricular function after phosphate supplementation is mainly observed in patients with severe hypophosphatemia (0.9 ± 0.15 mg/dL), but not in patients with mild hypophosphatemia (1.4 ± 0.11 mg/dL) or in the normal range. In contrast to these data, we noted an improvement in cardiorespiratory efficiency during exercise following SP supplementation in athletes within the physiological reference range of serum Pi levels (2.7–4.5 mg/dL). Similar results were also recorded in earlier studies in normoxia [7,9], which indicate that phosphate salts may be beneficial not only in subjects with low Pi serum level.

In our study, we recorded a significant (8.7%) increase in serum Pi concentrations after six-day SP supplementation. Similar results were reported by other authors [3,4,7,9,60,61], who observed an increase in blood Pi levels in the range of 5–39% after short term phosphate salt intake. There is evidence of a correlation between serum Pi concentration and Pi level in the myocardium [58]. Thus, it is likely that the administration of phosphate salts led to an increase in extracellular phosphate availability, promoting an increase in the intracellular phosphate level, thus enhancing ATP synthesis in the myocardium and improving myocardial contractility and SV in athletes.

Since the SV is also largely determined by blood volume [27,62], it is possible that an increase in SV after SP supplementation results from an increase in PV caused by an increase in serum sodium concentration [63,64]. During our experiment, blood sodium concentration was not determined. However, we did not observe changes in Hct and PV in cyclists after administration of phosphate salts. This is partially consistent with the results of previous study conducted by Marcos et al. [38], who reported no changes in Hct. Thus, it may be concluded that the increase in PV was not the mechanism responsible for the improvement in cardiovascular efficiency after SP supplementation in our study. 

It is worth noting that the short-term SP supplementation did not change blood Ca levels, which is consistent with previous reports [7,60]. A decrease in blood Ca concentration was registered after prolonged (four weeks) SP intake [7]. Calcium plays an essential role in several metabolic processes in the organism: it is a bone component and is also necessary for muscle contraction, nerve conduction, and blood coagulation [65]. Furthermore, intracellular calcium ions (Ca^2+^) are central regulators of cardiomyocyte contraction [66,67]. It was recognized that low blood Ca levels (hypocalcemia) are associated with neuromuscular irritability, muscle cramps, bone fragility, cognitive impairment, and cardiac symptoms, including prolonged QT intervals and cardiac arrhythmias [68]. Thus, the absence of disturbance in serum Ca levels after phosphate loading is favorable for athlete health and performance.

The second purpose of the present study was to investigate the effect of SP supplementation on mechanical efficiency in cyclists. We found that GE during exercise at the same absolute power output did not change after phosphate salt loading. When GE is considered for the same absolute power output, its improvement is strictly dependent on a reduction in VO_2_ and/or RER at this workload. In our study, we did not observe any significant changes in VO_2_ and RER during exercise at a given workload. Similarly, no significant decrease in submaximal RER or VO_2_ after phosphate loading was reported in studies conducted under normoxic conditions [10,38,44,69]. Our findings suggest that phosphate salts did not shift the use of metabolic substrates towards fat utilization and did not reduce the energy cost of exercise at an intensity > 60% of VO_2max_ in hypoxic conditions. Interestingly, Marcos et al. [38] found that SP ingested for seven days at a dose of 50 mg/kg lean body mass improved energy efficiency at low exercise intensity, where the major metabolic substrate for energy production is fat oxidation. It should be noted that efficiency in their study was calculated for a lower exercise intensity than in our experiment, which may be reflected by the differences in RER (0.82–0.86 vs. 0.93–0.98). These discrepancies may have contributed to the differences in the occurrence of efficiency improvement. The above observation suggests that SP supplementation may offer more ergogenic benefits for low-intensity exercise. Since hypoxia impairs aerobic metabolism [70], it is also possible that the energy cost of exercise is reduced following intake of phosphate salts when exercise is conducted under normoxic [38] but not hypoxic conditions. However, further investigation is needed to clarify this issue.

## 5. Practical Applications

It is considered that for ultraendurance events (i.e., >4 h), an optimal performance intensity exists below the anaerobic threshold [71,72]. Relative exercise intensity observed during ultraendurance competitions is around 60–75% of VO_2max_ in 4–12 h events [73,74,75], and decreases gradually during longer races [76,77]. In our study, we observed an ergogenic effect of SP on cardiovascular efficiency at workloads below LT, where the exercise intensity was <75% VO_2max_. Thus, we suggest that phosphate salt supplementation may be particularly beneficial during prolonged endurance exercise at altitude, for example during ultra-distance cycling, triathlon, running, and cross country skiing races. Future research should examine the effect of phosphate salt supplementation on exercise performance and physiological and biochemical responses during prolonged exercise protocols lasting several hours to determine phosphate loading suitability for ultraendurance athletes.

## 6. Study Limitations

To the best of our knowledge, our study is the first in which the effect of SP supplementation was assessed in normobaric hypoxia. Our experiment, however, is not without certain limitations. Firstly, during the experiment, subjects did not share the same accommodations and did not follow the same training schedule. However, they received nutritional, training, and supplementation recommendations that were constantly monitored. Secondly, SV and Q were estimated from ergospirometric measurements, which could have influenced the results. Future research should use more accurate methods to measure SV [78] and other indicators of myocardial efficiency. 

## 7. Conclusions

Short-term sodium phosphate supplementation promotes improvements in the efficiency of the cardiorespiratory system during low- and moderate-intensity exercise in a hypoxic environment without calcium-phosphate imbalance. Gross efficiency in hypoxia did not change following supplementation. We suggest that sodium phosphate should be considered as an ergogenic aid for endurance athletes, especially for ultra-race competitors. 

## Figures and Tables

**Table 1:** Exercise intensity presented as a percentage of actual maximal oxygen uptake.

Workload	Variable	Sodium Phosphate		Placebo	
		S1	S2	S1	S2
LT−2	% of actual VO_2max_	61.5 ± 1.3	59.9 ± 1.2	62.0 ± 1.3	62.7 ± 1.3
LT−1	% of actual VO_2max_	72.7 ± 1.1	72.0 ± 1.2	74.1 ± 1.2	74.7 ± 1.3
LT	% of actual VO_2max_	83.9 ± 0.9	82.3 ± 1.0	84.5 ± 0.9	85.6 ± 1.1
LT+1	% of actual VO_2max_	93.7 ± 0.7	92.0 ± 0.9	94.4 ± 0.8	94.9 ± 1.0
LT+2	% of actual VO_2max_	99.3 ± 0.5	96.6 * ± 0.8	97.3 ± 0.6	98.6 ± 1.0

LT−2, LT−1, LT, LT+1, LT+2—absolute workload corresponding to workload at lactate threshold (LT) as well as one and two workloads below and above LT which were determined during the initial exercise test (S1); S1—before supplementation phase, S2—after supplementation phase; * *p* < 0.05 S1 vs. S2.

**Table 2:** Stroke volume (SV), cardiac output (Q) and oxygen uptake (VO_2_) during exercise in hypoxia before (S1) and after (S2) sodium phosphate and placebo supplementation.

Workload	Variables	Sodium Phosphate		Placebo	
		S1	S2	S1	S2
LT−2	SV (mL)	123.6 ± 2.9	128.2 ± 3.6	120.8 ± 3.0	121.2 ± 3.7
	Q (L∙min^−1^)	15.95 ± 0.27	15.85 ± 0.32	16.15 ± 0.28	15.88 ± 0.38
	VO_2_ (L∙min^−1^)	2.12 ± 0.06	2.10 ± 0.06	2.17 ± 0.06	2.18 ± 0.06
LT−1	SV (mL)	117.9 ± 2.7	122.7 ** ± 3.1	117.4 ± 2.8	116.9 ± 3.2
	Q (L∙min^−1^)	17.10 ± 0.27	17.08 ± 0.32	17.36 ± 0.28	17.17 ± 0.32
	VO_2_ (L∙min^−1^)	2.51 ± 0.06	2.53 ± 0.07	2.59 ± 0.06	2.59 ± 0.07
LT	SV (mL)	113.8 ± 2.6	116.6 * ± 2.5	113.6 ± 2.6	113.3 ± 2.6
	Q (L∙min^−1^)	18.05 ± 0.27	17.97 ± 0.28	18.22 ± 0.28	18.19 ± 0.29
	VO_2_ (L∙min^−1^)	2.90 ± 0.06	2.89 ± 0.06	2.95 ± 0.06	2.97 ± 0.06
LT+1	SV (mL)	110.8 ± 2.4	112.3 ± 2.3	110.2 ± 2.5	110.6 ± 2.4
	Q (L∙min^−1^)	18.75 ± 0.29	18.69 ± 0.28	18.94 ± 0.30	18.93 ± 0.29
	VO_2_ (L∙min^−1^)	3.23 ± 0.06	3.22 ± 0.06	3.32 ± 0.07	3.30 ± 0.06
LT+2	SV (mL)	107.6 ± 3.0	109.2 ± 3.2	109.4 ± 3.5	109.4 ± 3.7
	Q (L∙min^−1^)	19.17 ± 0.40	19.10 ± 0.44	19.50 ± 0.47	19.48 ± 0.51
	VO_2_ (L∙min^−1^)	3.44 ± 0.06	3.38 ± 0.06	3.46 ± 0.07	3.43 ± 0.07

LT−2, LT−1, LT, LT+1, LT+2—absolute workload corresponding to workload at lactate threshold (LT) as well as one and two workloads below and above LT which was determined during the initial exercise test (S1). * *p* < 0.05, ** *p* < 0.01 S1 vs. S2.

**Table 3:** Gross efficiency (GE) and respiratory exchange ratio (RER) during exercise in hypoxia before (S1) and after (S2) sodium phosphate and placebo supplementation.

Workload	Variables	Sodium Phosphate		Placebo	
		S1	S2	S1	S2
LT−2	GE (%)	18.73 ± 0.47	18.96 ± 0.45	19.06 ± 0.50	19.00 ± 0.48
	RER	0.94 ± 0.01	0.93 ± 0.01	0.94 ± 0.01	0.93 ± 0.01
LT−1	GE (%)	20.20 ± 0.43	20.05 ± 0.41	20.54 ± 0.45	20.39 ± 0.43
	RER	0.97 ± 0.01	0.97 ± 0.01	0.98 ± 0.01	0.97 ± 0.01

LT−2, LT−1—absolute workload corresponding to one and two workloads below lactate threshold (LT) which was determined during the initial exercise test (S1).

## Data Availability

The data presented in this study are available on request from the corresponding author.
